# Solvent Extraction of Tellurium from Chloride Solutions Using Tri-n-butyl Phosphate: Conditions and Thermodynamic Data

**DOI:** 10.1155/2014/458705

**Published:** 2014-03-16

**Authors:** Dongchan Li, Yafei Guo, Tianlong Deng, Yu-Wei Chen, Nelson Belzile

**Affiliations:** ^1^Tianjin Key Laboratory of Marine Resources and Chemistry, Tianjin University of Science and Technology, Tianjin 300457, China; ^2^Engineering Research Center of Seawater Utilization Technology, Ministry of Education, Hebei University of Technology, Tianjin 300130, China; ^3^Department of Chemistry & Biochemistry, Laurentian University, Sudbury, ON, Canada P3E 2C6

## Abstract

The extractive separation of tellurium (IV) from hydrochloric acid media with tri-n-butyl phosphate (TBP) in kerosene was investigated. The dependence on the extraction of tellurium species, concentrations of tellurium and TBP, extraction time and stage, organic/aqueous ratio, and interferences from coexist metallic ions were examined and are discussed. Besides, the stripping agent and stripping time were also studied. It was found that the extraction reaction corresponds to the neutral complex formation mechanism and the extracted species is TeCl_4_ *·*3TBP and that the extraction process is exothermic. The thermodynamic parameters of enthalpy (Δ*H*), entropy (Δ*S*), and free energy (Δ*G*) of the extraction process were evaluated at −26.2 kJ*·*mol^−1^, −65.6 J*·*mol^−1^ *·*K^−1^, and −7.0 kJ*·*mol^−1^, respectively at 293 K.

## 1. Introduction 

Tellurium (Te) is widely used in various fields of human activity. As an excellent semiconductor of scattered elements, Te has unique applications in the electronic industry especially in the area of thermoelectricity, either for power generation as lead telluride or for refrigeration as bismuth telluride [1]. The other main application of Te is in metallurgy as an alloy with cast iron, copper, and stainless steel and when it is added to lead, it increases its strength and prevents corrosion [2]. It is also used in the rubber, glass, and ceramic industries. Meanwhile, Te is present in small amounts in the earth's crust with the average abundance of ~1 *μ*g·kg^−1^ (ppb). The proven and recoverable reserves of metallic Te are only ~38 000 tons in the world.

Due to this situation, extraction and separation of Te from complex samples are of great importance for the development of existing resources. Various solvent extraction methods have been used for the recovery of Te. The use of extractants tris-(2-ethyl hexyl) phosphate [3], tributyl phosphate (TBP) [4–6], 2,3,5-triphenyltetrazolium chloride [7], triphenylarsine oxide and tributyl-phosphine oxide [8], tri-isooctylamine [2], and* N*-*n*-octylaniline [1, 9] has been reported for the separation and extraction of Te. Extractant TBP [4–6] has been widely used for Te extraction due to its high efficiency, low consumption, and the simplicity of operation. The mechanism of Te separation from selenium in hydrochloric acid media with TBP in kerosene has been investigated in a batch-stirred glass cell. The extracted species and the extraction enthalpy were determined [4]. The mass transfer of Te(IV) between an aqueous and an organic phase has been studied in a modified Lewis cell with the organic phase being composed of TBP in kerosene at varying volume ratios [5]. A mass transfer model has been developed to indicate reaction front position and the extent of extraction of Te(IV) under different experimental conditions [6]. This study explored systematically the parameters affecting the extraction and stripping of Te, the extraction mechanism and allowed to estimate the thermodynamics factors of the overall extraction process.

## 2. Experiment

### 2.1. Reagents and Materials

All chemicals and solvents used in this study were of analytical reagent grade and the water was double distilled (DDW). The tri-butyl-phosphate (TBP) was supplied by Chengdu Kelong Corporation, China. It was diluted with aviation kerosene. The Te(IV) solution was prepared by dissolving the appropriate mass of potassium tellurite (K_2_TeO_3_) (Beijing Zhongliante Corporation, China) in 10% (v/v) hydrochloric acid and diluted to volume with DDW. The Te(VI) solution was prepared from sodium tellurate (Na_2_TeO_4_·2H_2_O) (Alfa Aesar, USA) in 10% (v/v) hydrochloric acid.

### 2.2. General Procedure

Aqueous solutions of Te(IV) were made with different concentrations of HCl to reach the required acidity. Organic solutions were prepared by diluting measured volumes of TBP with aviation kerosene. Then, these two solutions were transferred to a 125 mL separatory funnel according to selected phase ratios and the mixture was shaken for 3 min at 200 rpm as controlled by the HZQ-C rocking incubator (Harbin Donglian Electronic & Technology Development Co. Ltd., China). The two layers were allowed to settle and separate. The aqueous layer was discarded less than 1 minute. Tellurium(IV) from the aqueous phase was determined with hydride generation atomic florescence spectrometry (HG-AFS, model 2202, Beijing Haiguang Instrument Co. Ltd., China) with an uncertainty within 0.5% in mass [10]. Te concentrations in the organic phase were calculated from the different concentration values in the aqueous phase before and after the extraction. Then Te(IV) was stripped from the organic phase with 20% (w/v) ammonium chloride. The presence of coexisting metallic ions to the Te extraction process was measured by a Perkin-Elmer Optima 5300V ICP-OES with an uncertainty within 0.5% in mass.

## 3. Results and Discussion 

### 3.1. Choice of Diluents

The diluent plays a very important role in the solvent extraction process. It can change some physicochemical properties of the organic phase, such as density, viscosity, solubility parameter, and dipole moment, which affect the extraction efficiency. Moreover, the diluent itself cannot extract the metal to a significant quantity. On this basis, Chowdhury and Sanyal investigated the influence of diluents on the extraction of TeCl_4_ from the aqueous hydrochloric acid solution with TBP [11]. They divided the common diluents into two broad categories: polar and nonpolar. The result suggests that kerosene and hexane are the best and most competitive diluents for the system. In this study, we choose kerosene because of its low cost and minimum toxicity. It was also observed that the separation after the extraction process was quick and led to a clear phase.

### 3.2. Effect of Acid and Acid Concentration for Te(IV)

**Figure 1:**
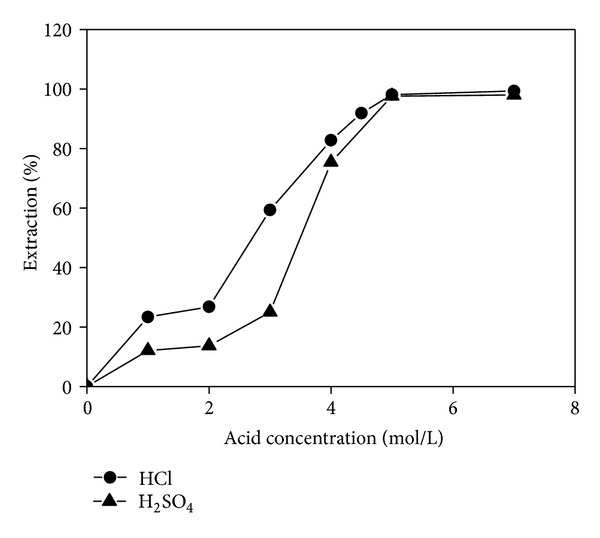
Effect of different concentrations of HCl and H_2_SO_4_ on Te(IV) extraction.

The extraction was carried out with an aqueous solution of 0.5 g/L Te(IV) and an organic solution containing 30% (v/v) TBP in kerosene at an organic/aqueous (O/A) volume ratio of 1. The effects of two acids sulfuric acid (H_2_SO_4_) and hydrochloric acid (HCl) on Te(IV) extraction were investigated. The results presented in Figure 1 indicate that the extraction of Te(IV) increased with the increasing concentration of both acids and that Te(IV) was almost completely extracted in 7 mol/L HCl and H_2_SO_4_. It was also found that the extraction efficiency started to be higher when the concentration of both acid was over 4 mol/L. However, in view of the serious corrosion effect of H_2_SO_4_, a 4.5 mol/L HCl was chosen for Te(IV) extraction in all additional experiments.

### 3.3. Extraction of Tellurium(VI)

The extraction was carried out with an aqueous solution of 0.5 g/L Te(VI) and an organic solution containing 30% (v/v) TBP in kerosene at an O/A ratio of 1 using 4.5 mol/L HCl. The mixture solution was shaken for 3 min at 200 rpm. The results (Table 1) show that Te(VI) was hardly extracted in the HCl medium with the low extraction efficiency. This suggests that Te(VI) does not interfere in the extraction of Te(IV) in 4.5 mol/L HCl, as it was carried out in this study.

### 3.4. Effect of Contact Time

**Figure 2:**
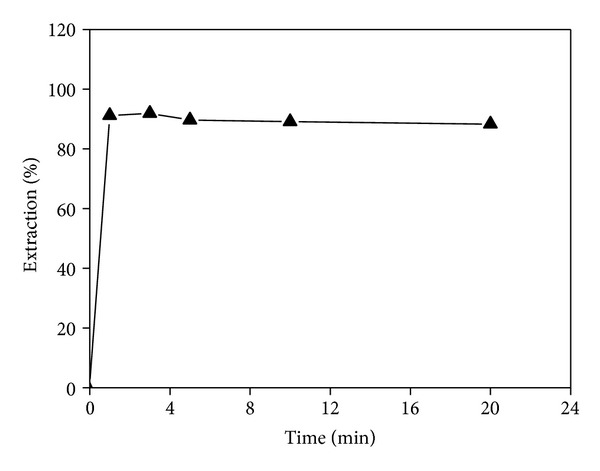
Effect of contact time on Te(IV) extraction.

The solution mixture of the two phases was shaken during periods varying from 1 to 20 min. The results show that the extraction was over 90.0% for 1 min and that the maximum extraction of 91.9% was obtained after 3 min of contact time. However, after 3 min, the extraction slightly decreased with increasing contact time (Figure 2). The extraction efficiency declined to 88.3% when the contact time was extended to 20 min. This may simply be due to the competitive extraction of aqueous phase and new extraction equilibrium established with the extension of time. A 3 min contact time is therefore recommended to reach the high extraction efficiency and ensure the optimum time of the metal ion extracted from the hydrochloric acid medium.

### 3.5. Effect of TBP Concentration in the Organic Phase

The effect of the concentration of extractant TBP in the organic phase was also examined. Data presented in Table 2 show an obvious increase in Te extraction as the extractant concentration increased. It was found that the extraction efficiency was much lower than 90% when the TBP concentration in kerosene was under 30% (v/v). In consideration of cost and consumption, a 30% (v/v) TBP concentration in kerosene was selected as the optimum condition even though the higher TBP concentrations of 40% and 50% showed a little higher extraction efficiency.

**Figure 3:**
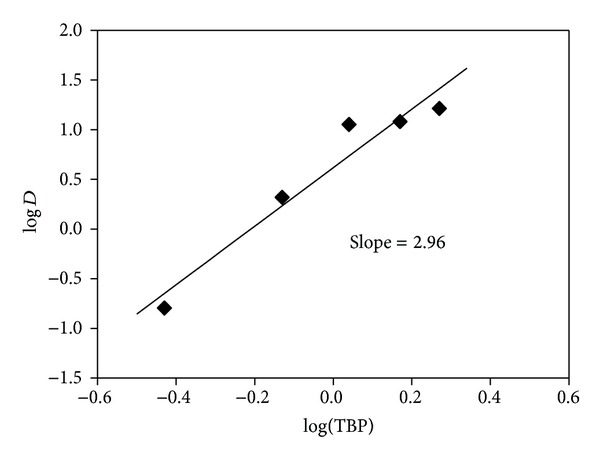
log⁡⁡*D* as a function of log TMP concentration.

The experiment also demonstrated that three TBP ligands react with one Te as revealed by the plot of log⁡⁡*D* versus log TBP. It generates a linear relationship with a slope very close to 3 at 2.96 (Figure 3).

Here are the corresponding equations [4]:

(1) $$\begin{array}{c} {\text{TeCl}}_{4(\text{aq})}+{m\text{TBP}}_{\left(\text{org}\right)}={\text{TeCl}}_{4}\cdot {m\text{TBP}}_{\left(\text{org}\right)}, \end{array}$$

extraction equilibrium (*K*_ex_):

(2) $$\begin{array}{c} K_{\text{ex}}=\frac{{\left[\text{TeC}{\text{l}}_{4}\cdot m\text{TBP}\right]}_{\text{org}}}{{\left[\text{TeC}{\text{l}}_{4}\right]}_{\text{aq}}{\left[\text{TBP}\right]}_{\text{org}}^{m}}, \end{array}$$

distribution coefficient (*D*):

(3) $$\begin{array}{c} D=\frac{{\left[\text{TeC}{\text{l}}_{4}\cdot m\text{TBP}\right]}_{\text{org}}}{{\left[\text{TeC}{\text{l}}_{4}\right]}_{\text{aq}}}. \end{array}$$

Therefore,

(4) $$\begin{array}{c} K_{\text{ex}}=\frac{D}{{\left[\text{TBP}\right]}_{\text{org}}^{m}}. \end{array}$$

For a constant [Cl^−^]

(5) $$\begin{array}{c} \text{Log}D=m\;\log{\left[\text{TBP}\right]}_{\text{org}}+\text{constant}. \end{array}$$

Figure 3 confirms that the value of the slope *m* is 2.96. The extraction reaction is therefore as follows:

(6) $$\begin{array}{c} {\text{TeCl}}_{4(\text{aq})}+3{\text{TBP}}_{\left(\text{org}\right)}={\text{TeCl}}_{4}\cdot 3{\text{TBP}}_{\left(\text{org}\right)}. \end{array}$$

### 3.6. Effect of Temperature

The effect of temperature on Te extraction from an aqueous phase of 0.5 g/L in 4.5 mol/L HCl with a 30% (v/v) TBP solution in kerosene was studied at 5 different temperatures. The results of Table 3 show that the extraction decreased with the increasing temperature.

The Van't Hoff equation shows the change on the distribution coefficient (*D*) with temperature and allows calculating the enthalpy of the Te extraction as

(7) $$\begin{array}{c} \frac{-\Delta H}{2.303R}=\frac{D\left(\log D\right)}{D\left(1/T\right)}. \end{array}$$

**Figure 4:**
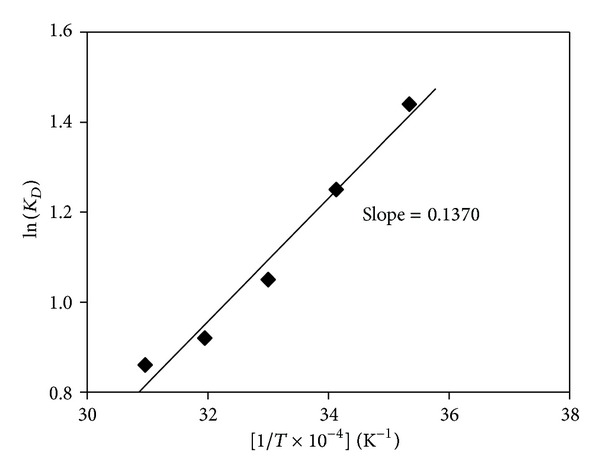
Effect of temperature on Te(IV) extraction.

The plot of log⁡⁡*D* versus 1/*T* × 10^−4^ (Figure 4) generates a linear relationship with a slope of 0.1370. The enthalpy change (Δ*H*) of the reaction was estimated at −26.23 kJ·mol^−1^, which indicates an exothermic reaction. This agrees well with a value of −26.8 kJ·mol^−1^ reported [4] for the same process under similar conditions.

The changes in free energy (Δ*G*) and entropy (Δ*S*) were then calculated using the following equations:

(8) $$\begin{array}{c} \Delta G=-2.303RT\;\log D,\\ \Delta S=\frac{\Delta H-\Delta G}{T}. \end{array}$$

The negative values of the free energy Δ*G* suggest that the reaction is favorable in nature. The negative enthalpy Δ*S* values indicate that the extraction is more favorable at low temperature (Table 4). Therefore, the ambient temperature range (283–303 K) can be considered as optimum for Te extraction. There are no values of Δ*G* or Δ*S* reported in the literature for that extraction process.

### 3.7. Loading Capacity of TBP in the Extraction

**Figure 5:**
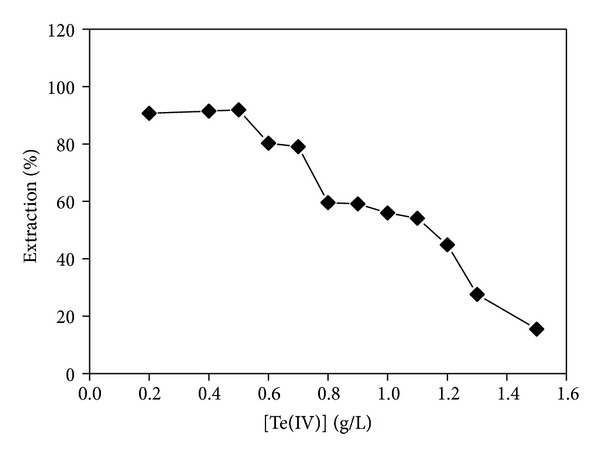
Effect of the initial concentrations of Te(IV) on extraction.

Aqueous solutions with various concentrations of Te were extracted at a fixed concentration of 30% (v/v) TBP in kerosene and 4.5 mol/L HCl. The extraction efficiency decreased sharply from 91.88% to 15.51% when the Te concentration in the aqueous solution exceeded 0.5 g/L (Figure 5). This result suggests that, to maintain a good efficient extraction, the concentration of TBP should be increased when that of Te gets higher than 0.5 g/L.

### 3.8. Effect of Organic to Aqueous Volume Ratio

Different volume ratios of organic to aqueous phase were tested and the results shown in Table 5 indicate that the extraction increased with an increasing O/A phase ratio, reaching a maximum value at 2 : 1. However, considering the sharp increase extraction when the O/A phase ratio was changed from 1 : 2 to 1 : 1, a preferred O/A phase ratio of 1 : 1 was adopted to avoid a large consumption of organic solution.

### 3.9. Effect of Repeated Extraction

The aqueous phase solution of 0.5 g/L Te(IV) in 4.5 mol/L HCl was extracted three times by the 30% (v/v) TBP solution in kerosene. Each equilibration time was 3 min. Results show that the extraction increased with the increasing number of extractions (Table 6). It reached up to a maximum of 98.97% after 3 extractions and this should be the preferred number of extractions for such a system.

### 3.10. Effect of Stripping Agents

Solutions of Te extracted into TBP in kerosene were submitted to different types of stripping agents. The mixture of the organic and stripping solutions were strongly shaken and then left to obtain a clear phase separation. The results (Table 7) show that stripping with water only was incomplete and inefficient in comparison with using a 20% (w/v) ammonium chloride solution, 1 mol/L sodium hydroxide, and 0.5 mol/L hydrochloric acid. Considering the high efficiency and the low corrosion effect of the ammonium chloride solution, it was chosen as the best stripping agent.

### 3.11. Effect of Stripping Time

**Figure 6:**
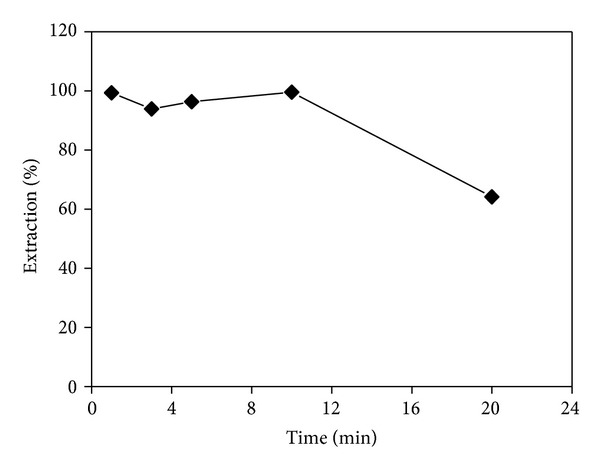
Effect of stripping time on Te(IV) extraction.

The effect of time on the stripping of Te was tested by shaking for different lengths of time using the 20% (w/v) ammonium chloride solution. As revealed in Figure 6, Te in the organic phase was almost stripped completely within 1 min but the extraction efficiency showed some fluctuations with extended time. A 10 min stripping time proved to be the most efficient with 99.39%, but it went sharply down to 64.19% when the time was prolonged to 20 min, since a very high stripping efficiency and a clear phase separation could be obtained within only 1 min, which time was chosen for stripping Te.

### 3.12. Effect of Diverse Ions

Finally, the effect of diverse common ions on the recovery of Te was investigated by adding different amounts of selected ionic species (K^+^, Na^+^, Ca^2+^, Mg^2+^, Fe^3+^, Cu^2+^, and Mn^2+^). The amount of each ionic species added to the extraction system before extraction (taken amount) and after extraction (determined amount) was examined. The data in Table 8 indicate that these common ions do not interfere with the extraction of Te in 4.5 mol/L HCl with 30% (v/v) TBP in kerosene at 1 : 1 O/A ratio.

## 4. Conclusion

Different parameters were investigated for the extraction of tellurium species from an acid medium. In consideration of cost, time, use of solvent, and overall efficiency, it was determined that the extraction of an aqueous solution of 0.5 g/L Te(IV) was the best in 4.5 mol/L HCl with an organic solution containing 30% (v/v) TBP in kerosene at an organic/aqueous (O/A) volume ratio of 1, with a contact time of 3 min and 3 repeated extractions. The Te extraction in such system can reach a 99% recovery. The stripping agent and stripping time were also looked into for Te(IV) from the organic solution and a 20% (w/v) ammonium chloride solution was chosen with a stripping time of 1 min and the stripping rate was up to 99%. Further, based on the experiment data, the extraction reaction was found to be neutral complex mechanism, and the extracted species in the organic phase is TeCl_4_·3TBP. The extraction efficiency decreased with the increasing temperature. The extraction reaction is an exothermic process. The thermodynamic functions of enthalpy (Δ*H*), entropy (Δ*S*), and free energy (Δ*G*) of the Te extraction with TBP have been evaluated at the chosen conditions.

## Figures and Tables

**Table 1:** Effect of HCl concentration on Te(VI) extraction.

HCl/(mol·L^−1^)	Extraction/%
1	0.61
3	0.62
5	2.98

**Table 2:** Effect of TBP concentration on Te(IV) extraction.

TBP/% (v/v)	*C*_TBP_/(mol·L^−1^)	log⁡⁡C_TBP_	Extraction/%	*D*	log⁡⁡D
10	0.37	−0.43	14.03	0.16	−0.79
20	0.74	−0.13	67.54	2.08	0.32
30	1.11	0.04	91.88	11.32	1.05
40	1.47	0.17	92.37	12.11	1.08
50	1.84	0.27	94.25	16.39	1.21

**Table 3:** Effect of temperature on Te(IV) extraction.

*T*/(K)	1/*T* × 10^−4^/(K^−1^)	Extraction/%	*D*	log⁡⁡D
283	35.34	96.51	27.65	1.44
293	34.13	94.64	17.66	1.25
303	33.00	91.88	11.32	1.05
313	31.95	89.22	8.28	0.92
323	30.96	87.82	7.21	0.86

**Table 4:** Values of Δ*G* and Δ*S* of the extraction process at different temperatures.

*T*/(K)	log⁡⁡D	Δ*G*/(kJ·mol^−1^)	Δ*S*/(J·mol^−1^·K^−1^)
283	1.44	−7.80	−65.12
293	1.25	−7.01	−65.60
303	1.05	−6.09	−66.47
313	0.92	−5.51	−66.20
323	0.86	−5.31	−64.77

**Table 5:** Effect of the volume phase ratio on Te(IV) extraction.

O/A/(*V*_*o*_/*V*_*w*_)	Extraction of Te/%
1 : 2	77.79
1 : 1	91.88
2 : 1	96.27

**Table 6:** Effect of repeated extraction on Te(IV) extraction.

Stage	Extraction of Te (additive)/%
1	91.88
2	97.76
3	98.97

**Table 7:** Effect of the stripping agent on Te(IV) extraction.

Agent	Extraction of Te/%
H_2_O	73.70
20% (w/v) NH_4_Cl	99.56
1 mol/L NaOH	98.93
0.5 mol/L HCl	93.10

**Table 8:** Effect of diverse ions on Te(IV) extraction.

Ions	Amount taken/(mg·L^−1^)	Amount determined/(mg·L^−1^)
K^+^	9.9	9.3
Na^+^	6.5	6.5
Ca^2+^	9.3	9.0
Mg^2+^	5.8	5.7
Fe^3+^	2.9	3.2
Cu^2+^	11.2	11.8
Mn^2+^	11.0	11.7
